# Postnatal Overnutrition Induces Changes in Synaptic Transmission to Leptin Receptor-Expressing Neurons in the Arcuate Nucleus of Female Mice

**DOI:** 10.3390/nu12082425

**Published:** 2020-08-13

**Authors:** Thais Tessari Zampieri, Tabata Mariz Bohlen, Marina Augusto Silveira, Larissa Campista Lana, Daniella G. de Paula, Jose Donato, Renata Frazao

**Affiliations:** 1 Department of Anatomy, Institute of Biomedical Sciences, University of São Paulo, 05508-000 São Paulo, SP, Brazil; thaizampieri@gmail.com (T.T.Z.); bohlen.tabata@gmail.com (T.M.B.); masilveira89@gmail.com (M.A.S.); larissa.campista@gmail.com (L.C.L.); daniella.gpaula@gmail.com (D.G.d.P.); 2 Department of Physiology and Biophysics, Institute of Biomedical Sciences, University of São Paulo, 05508-000 São Paulo, SP, Brazil; jdonato@icb.usp.br

**Keywords:** hypothalamus, development, overweight, puberty

## Abstract

The adipocyte-derived hormone leptin is a potent neurotrophic factor that contributes to the neural plasticity and development of feeding circuitry, particularly in the arcuate nucleus of the hypothalamus (ARH). Postnatal overnutrition affects leptin secretion and sensitivity, but whether postnatal overnutrition produces changes in the development of the synaptic transmission to ARH neurons is currently unknown. We evaluated the excitatory and inhibitory currents to ARH leptin receptor (LepR)-expressing neurons in prepubertal, pubertal and adult female mice. The effects of postnatal overnutrition in the expression of genes that code ion channels subunits in the ARH were also evaluated. We observed that the transition from prepubertal to pubertal stage is characterized by a rise in both excitatory and inhibitory transmission to ARH LepR-expressing neurons in control mice. Postnatal overnutrition induces a further increase in the excitatory synaptic transmission in pubertal and adult animals, whereas the amplitude of inhibitory currents to ARH LepR-expressing cells was reduced. Postnatal overnutrition also contributes to the modulation of gene expression of N-methyl-D-aspartate, GABA_B_ and ATP-sensitive potassium channel subunits in ARH. In summary, the synaptic transmission to ARH cells is profoundly influenced by postnatal overnutrition. Thus, increased adiposity during early postnatal period induces long-lasting effects on ARH cellular excitability.

## 1. Introduction

Postnatal overnutrition is a risk factor for metabolic disorders such as obesity, type 2 diabetes and cardiovascular diseases [1,2,3]. Brain circuitry development is profoundly influenced by postnatal nutritional variations, mainly because of the action of the adipocyte-derived hormone leptin [4]. Leptin is considered a potent neurotrophic factor to promote the development of the feeding circuits in the arcuate nucleus of the hypothalamus (ARH) [5,6,7]. This neurotrophic effect is important because ARH is considered the main mediator of leptin’s effects on energy homeostasis [8,9]. In addition to feeding regulation, ARH neurons also act as a neuroendocrine integrator and play a central role in reproduction, among other functions [10,11]. The ARH is composed of diverse classes of neurons distributed along its rostro-caudal extension. The neurochemical identity of ARH neurons include cells that produce a large diversity of neurotransmitters, including GABA, glutamate, neuropeptide Y (NPY), agouti-related peptide (AgRP), pro-opiomelanocortin (POMC) and kisspeptin [12,13]. Several neural populations of the ARH express the long isoform of LepR which is responsible for the major biological effects of leptin [12,13].

Hypothalamic neurons are constantly exhibiting neuroplasticity in response to homeostatic demands [14,15,16,17,18,19,20]. For example, GABAergic inputs to NPY or POMC neurons increase from prepubertal to adult stage, coinciding with the development of feeding circuitry in mice [5,16,21]. In adult mice, diet-induced obesity suppresses GABAergic and glutamatergic tone to NPY neurons, whereas fasting increases excitatory transmission to NPY-expressing cells [16,18,19,20,22]. On the other hand, postnatal undernutrition negatively influences GABAergic and glutamatergic transmission to NPY cells in male and female mice at an early age, P30-33 [23]. However, whether postnatal overnutrition is sufficient to induce long-term effects on excitatory synaptic plasticity of ARH neurons is unknown.

To determine whether postnatal overnutrition influences the neuroplasticity of ARH neurons, electrophysiological experiments and mRNA analysis were performed. A postnatal overnutrition model by raising mice in small litters (SL) was employed and compared to animals raised in normal size litters (control). The excitatory and inhibitory synaptic transmission to ARH LepR-expressing cells were evaluated at prepubertal, pubertal and adult female mice. The gene expression of several ion channels subunits was also evaluated in prepubertal and adult mice. Our findings indicate that postnatal overnutrition led to significant age-dependent changes in synaptic transmission to ARH LepR-expressing cells. In addition, postnatal overnutrition affected the expression of genes coding for N-methyl-D-aspartate, GABA_B_ and ATP-sensitive potassium channel (K_ATP_) subunits in the ARH, indicating that postnatal overnutrition induces long-lasting effects on ARH neurons activity.

## 2. Materials and Methods

### 2.1. Animals

LepR-IRES-Cre mice (C57BL/6J, Stock No: 008320, The Jackson Laboratory, Bar Harbor, ME, USA) were crossed with the Lox-Stop-Lox (LSL) Cre-inducible tdTomato-reporter mice (C57BL/6J, Stock No: 007909, The Jackson Laboratory). The LepR-expressing cells were identified via tdTomato fluorescent protein expression [24]. The LepR reporter mice were housed in the animal care facility of the Department of Anatomy in the Institute of Biomedical Sciences at the University of São Paulo, with controlled light (12 h on/12 h off; lights on at 0600 h) and temperature (23 ± 2 °C). All experiments and procedures were approved by the Institutional Animals Ethics Committee of the Institute of Biomedical Sciences at the University of São Paulo (Protocol # 21/2017), and performed in accordance with the guidelines established by the National Institute of Health’s Guide for the Care and Use of Laboratory Animals.

### 2.2. Experimental Design

Evaluation of sexual maturation: To determine the effects of postnatal overnutrition on synaptic transmission of ARH LepR-expressing cells, mice were raised in SL, as previously described [25,26]. Briefly, litter sizes were modified on postnatal day 5, with 3 pups per litter in the SL group, and 7–8 pups per litter in the control group. Female mice were selected for experiments according to their age: prepubertal (8–12 days), pubertal (38–42 days) and adult (60–90 days). Female mice were selected for experiments because sexual maturation progression can be determined in vivo. To study pubertal and adult mice, we evaluated sexual maturation, as previously described [25,27,28]. In this regard, we recorded the age of vaginal opening and the first occurrence of vaginal cornification in the vaginal lavage (first estrus). After detecting regular estrous cyclicity, we selected adult females in diestrus for the experiments. The body weight was recorded for prepubertal, pubertal and adult control and SL mice. Mice were anesthetized and sacrificed. Blood samples and subcutaneous fat pad were collected for subsequent analysis and the brain was used for electrophysiological experiments. Serum leptin levels were determined using a commercially available enzyme-linked immunosorbent assay kit (Crystal Chem, Elk Grove Village, IL, USA), according to the instructions of the manufacturer. Leptin kit has a detection limit of 0.2 ng/mL and an intra- and inter-assay coefficient of variability ≤10%. 

### 2.3. Voltage-Clamp Recordings

To characterize spontaneous currents of ARH LepR-expressing neurons, we performed whole-cell voltage-clamp recordings. The hypothalamic slices were obtained from prepubertal, pubertal and adult female LepR reporter mice. Brains were rapidly removed from anesthetized animals and hypothalamic slices (200 µm for prepubertal; 250 μm for pubertal and adult) were processed, as previously described [29]. Most of recordings were performed in LepR-expressing cells located in the ventromedial aspects of the ARH (130 μm of maximal distance from the median eminence and third ventricle). Spontaneous excitatory postsynaptic currents (sEPSC) were recorded in LepR-expressing neurons at voltage clamp at −65 mV. Slices were maintained in artificial cerebral spinal fluid (ACSF) containing: 124 mM NaCl, 2.8 mM KCl, 26 mM NaHCO_3_, 1.25 mM NaH_2_PO_4_, 1.2 mM MgSO_4_, 5 mM glucose and 2.5 mM CaCl_2_. The pipette solution was composed of: 120 mM K-gluconate, 1 mM NaCl, 10 mM KCl, 10 mM HEPES, 5 mM EGTA, 1 mM CaCl_2_, 1 mM MgCl_2_, 3 mM KOH and 4 mM (Mg)-ATP, pH 7.3 (E_CL_ = −57 mV). When filled, the pipettes had a resistance of 5–7 MΩ. When ACSF contained CNQX and AP5 were used, no fast synaptic transmission was detected (data not shown), suggesting that ionotropic glutamate receptors account for the majority of the observed fast sEPSC. Spontaneous inhibitory postsynaptic currents (sIPSC) of LepR-expressing neurons were recorded at voltage-clamp mode at holding potential −60 mV. To increase the driving force for chloride ions, sIPSC recordings were made with an isotonic chloride pipette solution composed of: 140 mM KCl, 10 mM HEPES, 5 mM EGTA, 0.1 mM CaCl_2_, 8.5 mM NaOH, 4 mM (Mg)-ATP, 0.4 mM (Na)-GTP, pH 7.3 (E_CL_ = 0 mV). Therefore, the direction of net current flow was expected to be inward. Picrotoxin blocked most of fast synaptic transmission suggesting that GABA_A_ receptors account for the majority of the observed fast PSC observed with this solution (not showed). Capillaries had a resistance of 3-4.5 MΩ when filled with internal solution. During the recordings, neurons were maintained in hypothalamic slice preparation and electrophysiological signals were recorded using an Axopatch 700B amplifier (Molecular Devices, San Jose, CA, USA; RRID: SCR_011323), low-pass filtered at 2–4 kHz. Patch electrodes were pulled from borosilicate glass capillaries. The sEPSC and sIPSC currents were recorded for 2 min and analyzed using the Mini Analysis Program (Synaptosoft, Decatur, GA, USA; RRID: SCR_002184). Series resistance was <20 MΩ and data was discarded if a change of 20% or more occurred during the recording. Importantly, the series resistance and cell capacitance were similar between different groups and ages (data not showed). All events were detected with a threshold that was 5× the root-mean-square of the baseline noise and were re-examined manually before data acceptance. 

### 2.4. ARH mRNA Expression

The ARH mRNA expression analysis was performed using samples of prepubertal and adult female mice (second day of diestrus) from control and SL groups. Mice were anesthetized and decapitated, and the entire brain was removed. Coronal sections (500 µm) of the brain were obtained using a vibratome (Leica Biosystems, Buffalo Grove, IL. USA; RRID: SCR_016495). ARH micropunches were obtained from hypothalamic sections approximately at −1.44 to −1.94 mm from bregma [29]. Total RNA from the micropunches was extracted with the PicoPure RNA isolation kit (Thermo Fisher Scientific Cat# KIT0204, Waltham, MA USA; RID: SCR_008817), according to the instructions of the manufacturer. Assessment of RNA quantity and quality was performed with an Epoch Microplate Spectrophotometer (Gen5, BioTek, Winooski, VT, USA; RRID: SCR_017317). Total RNA was incubated with RNase-free DNase I (Roche, Welwyn Garden City, UK; RRID: SCR_001326). Reverse transcription was performed with 0.5 µg of total RNA, SuperScript^®^ II Reverse Transcriptase (Thermo Fisher Scientific) and random primers p(dN)6 (Roche). Real-time quantitative polymerase chain reaction (RT-qPCR) was performed using the 7500 Real-Time PCR System (Thermo Fisher Scientific) and optimized using Power SYBR Green PCR Master Mix (Thermo Fisher Scientific). Each target gene was evaluated in duplicate. Data were normalized by *Gapdh* expression and reported as fold change when compared to values obtained from the control group: (i.e., adult, set to 1.0). The primers utilized for the gene expression analyses are provided in Table 1. Melt curve analysis was conducted to validate the specificity of the primers. Relative quantification of mRNA was calculated by 2^−ΔΔCt^.

### 2.5. Statistical Analysis

Statistical analysis was performed using GraphPad Prism software (GraphPad Prism, San Diego, CA, USA; RRID: SCR_002798). The results are expressed as the mean ± standard error of the mean (SEM). The Mann–Whitney test was used to evaluate puberty events between mice obtained from SL to control animals. Differences between ages in the control group were evaluated using unpaired one-way ANOVA with Bonferroni’s post hoc test. Two-way ANOVA and Bonferroni’s post hoc test were used to compare SL to control mice and evaluate body weight, serum leptin levels, sEPSC and sIPSC frequency and amplitude. Results with a *p* value of <0.05 were considered to be statistically significant.

## 3. Results

### 3.1. Excitatory and Inhibitory Synaptic Transmission to ARH LepR-Expressing Cells Increase at Pubertal Stage

**Figure 1:**
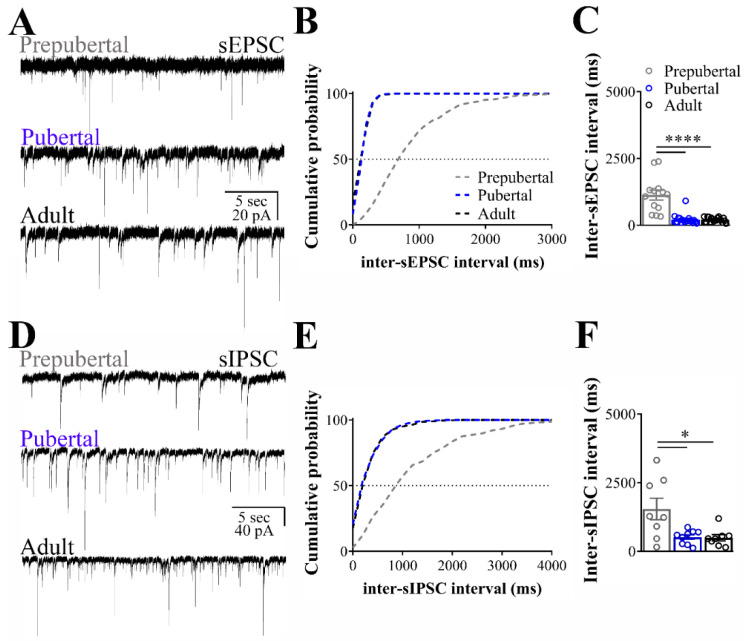
Spontaneous excitatory and inhibitory transmission to leptin receptor (LepR)- expressing neurons located at the arcuate nucleus (ARH). (**A**,**D**) Representative voltage-clamp recordings demonstrating the spontaneous postsynaptic currents (sEPSC, **A**) and the spontaneous inhibitory postsynaptic currents (sIPSC, **D**) recorded from ARH neurons of prepubertal, pubertal and adult female mice. (**B**,**C**,**E**,**F**) Cumulative probability histogram and inter-event interval bar graph demonstrating that prepubertal animals exhibited increased interevent interval of sEPSC (inter-sEPSC, **B**,**C**) and interevent interval of sIPSC (inter-sIPSC, **E**,**F**) compared to pubertal and adult mice. Pubertal and adult mice had similar cumulative probability distribution (traces are overlapped). One-way ANOVA followed by the Bonferroni post hoc test was used to evaluate data. All results were expressed as mean ± SEM. **** *p* < 0.0001, * *p* < 0.05.

To determine the excitatory transmission pattern of ARH LepR-expressing cells during development, we clamped the membrane potential at −65 mV. We recorded the sEPSC in cells from prepubertal, pubertal and adult female mice. A significant increase in sEPSC frequency was observed in pubertal and adult animals, compared to prepubertal mice (prepubertal: 0.9 ± 0.2 Hz; pubertal: 4.4 ± 0.5 Hz; adult: 4.0 ± 0.5 Hz; 13/19 cells out of four mice per group, F_(2, 48)_ = 175.59, *p* < 0.0001; Figure 1A). Consequently, the interval between excitatory synaptic currents (inter-sEPSC interval) decreased in pubertal and adult animals, compared to prepubertal mice (F_(2, 48)_ = 32.08, *p* < 0.0001, Figure 1B,C). On the other hand, sEPSC amplitudes were similar in all stages of development (prepubertal: 28.4 ± 0.8 pA; pubertal: 26.8 ± 0.6 pA; adult: 28.7 ± 1.3 pA; F_(2, 48)_ = 1.238, *p* = 0.2989).

In the next set of experiments, we clamped the membrane potential of ARH LepR-expressing neurons at −60 mV, and the sIPSC were recorded. The sIPSC frequencies were significantly greater in pubertal and adult animals when compared to prepubertal mice (prepubertal: 0.5 ± 0.1 Hz; pubertal: 1.8 ± 0.5 Hz; adult: 1.9 ± 0.4 Hz; 8/9 cells out of 4/6 mice per group, F_(2, 22)_ = 3.708, *p* = 0.0410; Figure 1D). Consistent with the increased average frequency, a decreased inhibitory interevent interval (inter-sIPSC interval) was identified by comparing prepubertal to pubertal and adult animals (F_(2, 22)_ = 6.491, *p* = 0.0061; Figure 1E,F). No differences in the amplitude of the sIPSC were detected between the different ages (prepubertal: 55.4 ± 4.2 pA; pubertal: 65.1 ± 5.2 pA; adult: 57.2 ± 6.7 pA; F_(2, 22)_ = 0.9130, *p* = 0.4160). These findings indicate that the transition from prepubertal to pubertal stage is characterized by an increase in the excitatory and inhibitory transmission to ARH LepR-expressing neurons.

### 3.2. Metabolic Consequences of the SL Model

To confirm the SL as a model that causes postnatal overweight, body weight, subcutaneous fat pad mass, serum leptin levels and the timing of puberty were evaluated in SL mice and in animals raised in normal size litters (control group) (Table 2). The SL mice exhibited increased body weight, higher subcutaneous fat pad mass and serum leptin levels at the prepubertal stage compared to control mice (Table 2). At puberty, body weight of SL mice was increased compared to control mice, despite no differences in subcutaneous fat pad mass or serum leptin levels. At adulthood, SL mice exhibited similar body weight, subcutaneous fat pad mass and serum leptin levels compared to control mice (Table 2). In accordance with previous studies [25,30], SL mice displayed early onset of puberty as demonstrated by earlier age of vaginal opening and first estrus, compared to control animals (Table 2).

### 3.3. Postnatal Overnutrition Amplifies the Excitatory Synaptic Transmission to ARH LepR-Expressing Neurons Starting at Puberty

**Figure 2:**
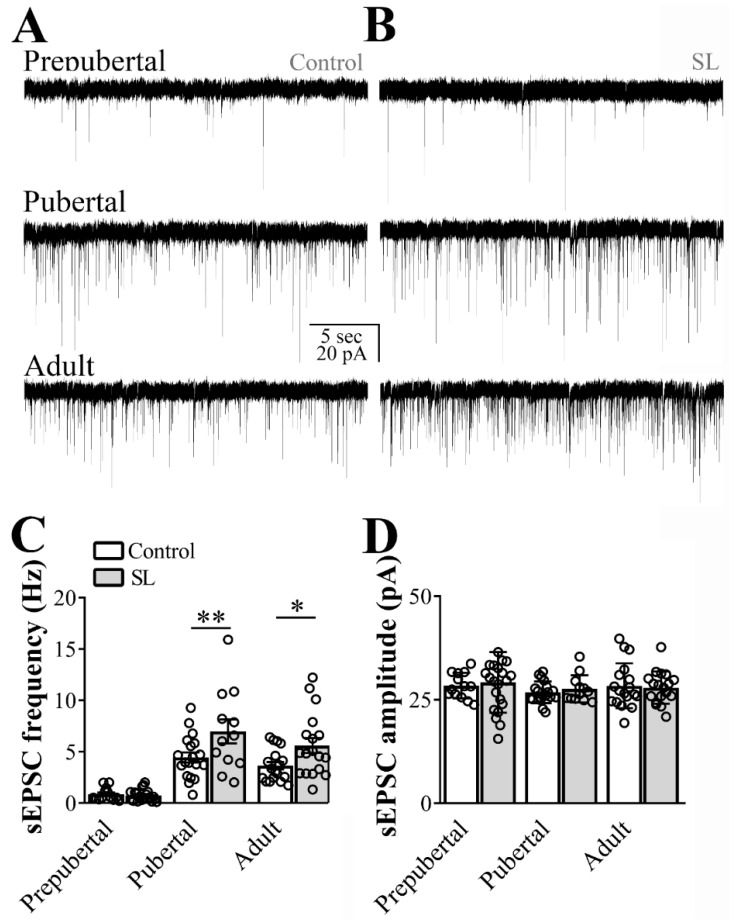
Postnatal overnutrition effects on excitatory transmission to leptin receptor (LepR)-expressing neurons located at the arcuate nucleus (ARH). (**A**,**B**) Representative voltage-clamp recordings demonstrating the spontaneous excitatory postsynaptic currents (sEPSC) of ARH neurons recorded from prepubertal, pubertal and adult control (**A**) or small litter (SL) female mice (**B**). (**C**,**D**) Bar graphs demonstrating the average frequency (**C**) and the average amplitude of the sEPSC comparing control to the SL group. Two-way ANOVA followed by the Bonferroni post hoc test was used to evaluate data. All results were expressed as mean ± SEM. An interaction between postnatal overnutrition and main age effect for sEPSC frequency were observed (F_(2, 95)_ = 3.164, *p* = 0.0468). All results were expressed as mean ± SEM. * *p* < 0.05, ** *p* ≤ 0.007.

To determine the effects of postnatal overnutrition on synaptic transmission, we recorded hypothalamic slices obtained from SL and control mice. Although sEPSC frequency was similar between SL and control mice at the prepubertal stage, postnatal overnutrition led to a significant increase in the sEPSC frequency in pubertal mice (main effect of litter size: F_(1, 96)_ = 10.46, *p* = 0.0017; main effect of age: F_(2, 96)_ = 43.57, *p* < 0.0001, *n* = 12/22 cells out of four mice per group, two-way ANOVA, followed by the Bonferroni post hoc test, Figure 2A,C). Importantly, the increased sEPSC frequency in ARH LepR-expressing cells of SL mice was still observed in adult animals. Consequently, a significant interaction between postnatal overnutrition and the main effect of age was noted (F_(2, 96)_ = 3.242, *p* = 0.0434), suggesting a long-lasting effect induced by postnatal overnutrition. No detectable effect of postnatal overnutrition on sEPSC amplitude was observed (litter size: F_(1, 96)_ = 0.1489, *p* = 0.7005, age: F_(2, 96)_ = 0.8540, *p* = 0.4289, interaction: F_(2, 96)_ = 0.1893, *p* = 0.8279, Figure 2D).

### 3.4. Inhibitory Transmission to ARH Neurons is Attenuated in SL Mice

**Figure 3:**
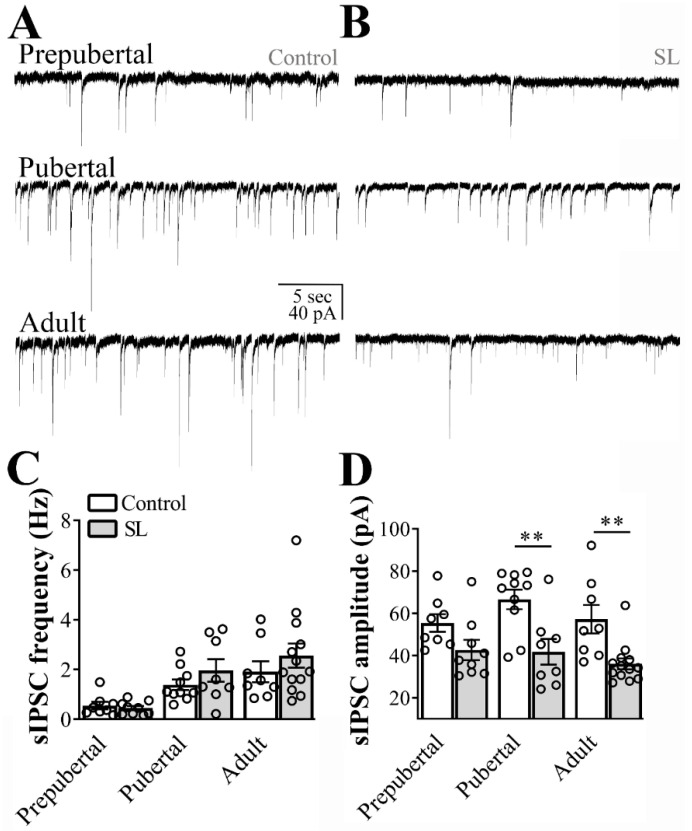
Postnatal overnutrition effects on inhibitory transmission to leptin receptor (LepR)- expressing neurons located at the arcuate nucleus (ARH). (**A**,**B**) Representative voltage-clamp recordings demonstrating the spontaneous inhibitory postsynaptic currents (sIPSC) of ARH neurons recorded from prepubertal, pubertal and adult control (**A**) or small litter (SL) female mice (**B**). (**C**,**D**) Bar graphs demonstrating the average frequency (**C**) and the average amplitude of the sIPSC comparing data from control to the SL group. Two-way ANOVA followed by the Bonferroni post hoc test was used to evaluate data. All results were expressed as mean ± SEM. ** *p* < 0.005.

Next, we recorded the spontaneous inhibitory transmission to ARH LepR-expressing neurons in hypothalamic slices obtained from SL mice. By comparing SL to control mice no detectable difference in the frequency of inhibitory currents to ARH LepR-expressing neurons was observed (main effect of litter size: F_(1, 50)_ = 1.467, *p* = 0.2315, main effect of age: F_(2, 50)_ = 11.03, *p* = 0.0001 and interaction: F_(2, 50)_ = 0.5864, *p* = 0.5601, *n* = 8/13 cells out of 4/6 mice per group, Figure 3C). Notably, the sIPSC amplitude was significantly reduced in SL mice in comparison to control animals (main effect of litter size: F_(1, 50)_ = 25.25, *p* < 0.0001, main effect of age: F_(2, 50)_ = 1.330, *p* = 0.2736 and interaction: F_(2, 50)_ = 0.8011, *p* = 0.4545, Figure 3D).

### 3.5. The Gene Expression of Several Ion Channel Receptors in the ARH is Modulated by Postnatal Overnutrition

**Figure 4:**
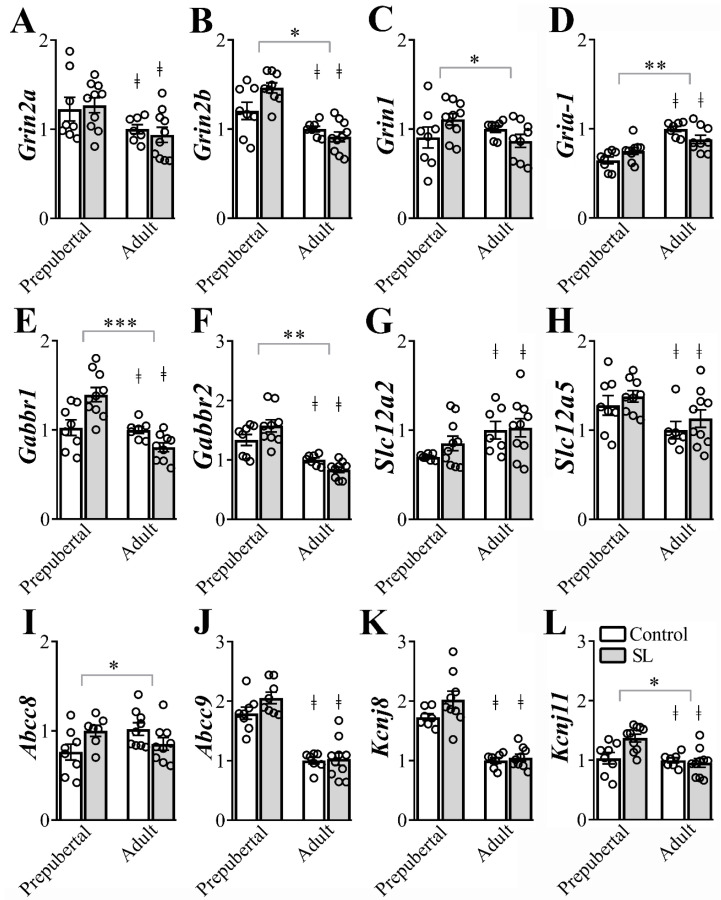
Postnatal overnutrition effects in the expression of genes coding for ion channels receptors in the arcuate nucleus (ARH). (A-D) ARH mRNA expression of *Grin2a* (**A**), *Grin2b* (**B**) *Grin1* (**C**) and *Gria-*1 (**D**), coding for glutamate ionotropic receptors. (**E**,**F**) ARH mRNA expression of the *Gabbr1* (**E**) and *Gabbr2* (**F**) coding for the GABA_B_ receptor. (**G**,**H**) ARH mRNA expression of *Slc12a2* (**G**) and *Slc12a5* (**H**), coding for the Na^+^-K^+^-Cl^-^- cotransporter type 1 and 2. (**I**,**L**) The mRNA levels of *Abcc8* (**I**), *Abcc9* (**J**), *Kcnj8* (**K**) and *Kcnj11* (**L**), coding for ATP-sensitive potassium channel, in the ARH. Two-way ANOVA followed by the Bonferroni post hoc test was used to evaluate data. All results were expressed as mean ± SEM. ^‡^ indicates main age effect. * indicates significant interaction between postnatal overnutrition and main age effects. *** *p* <0.0001, ** *p* ≤0.006, * *p* < 0.05.

Glutamate, GABA, Na^+^-K^+^-Cl^−^- cotransporter and K_ATP_ channels subunits play a key role in neuronal plasticity and dendritic development and some receptor subunits are expected to suffer age-dependent changes [15,31,32,33]. Therefore, we determined the gene expression of ion channels subunits in ARH micropunches from prepubertal and adult female mice (6/10 per group, Figure 4). Adult mice exhibited a significant reduction of *Grin2a* (F_(1, 31)_ = 8.650, *p* = 0.0061, Figure 4A), *Grin2b* (F_(1, 30)_ = 33.01, *p* < 0.0001, Figure 4B), *Gabbr1* (F_(1, 30)_ = 19.01, *p* = 0.0001, Figure 4E), *Gabbr2* (F_(1, 29)_ = 46.54, *p* < 0.0001, Figure 4F), *Slc12a5* (F_(1, 29)_ = 7.580, *p* = 0.0101, Figure 4H), *Abcc9* (F_(1, 29)_ = 88.32, *p* < 0.0001, Figure 4J), *Kcnj8* (F_(1, 28)_ = 80.19, *p* < 0.0001, Figure 4K), and *Kcnj11* (F_(1, 31)_ = 9.305, *p* = 0.0047, Figure 4L) mRNA expression compared to prepubertal mice. On the other hand, *Gria-1* (F_(1, 29)_ = 35.02, *p* < 0.0001, Figure 4D) and *Slc12a2* (F_(1, 30)_ = 7.133, *p* = 0.0121, Figure 4G) mRNA levels increased in the ARH of adult animals, compared to prepubertal mice. No significant age-dependent changes were observed in *Grin1* (F_(1, 30)_ = 0.8472, *p* = 0.3647, Figure 4C), *Gabra1* (F_(1, 29)_ = 0.6419, *p* = 0.4296) and *Abcc8* (F_(1, 29)_ = 0.2328, *p* = 0.6331, Figure 4I) mRNA levels in the ARH. 

Regarding the effects of postnatal overnutrition in the regulation of gene expression in the ARH, we observed a statistically significant interaction between postnatal overnutrition and age effects on *Grin2b* (F_(1, 30)_ = 6.943, *p* = 0.0132, Figure 4B), *Grin1* (F_(1, 30)_ = 4.410, *p* = 0.0442, Figure 4C), *Gria-1* (F_(1, 29)_ = 7.652, *p* = 0.0098, Figure 4D), *Gabbr1* (F_(1, 30)_ = 15.99, *p* = 0.0004, Figure 4E), *Gabbr2* (F_(1, 29)_ = 6.583, *p* = 0.0157, Figure 4F), *Abcc8* (F_(1, 29)_ = 7.375, *p* = 0.0110, Figure 4I) and *Kcnj11*(F_(1, 31)_ = 6.649, *p* = 0.0149, Figure 4L) mRNA levels in the ARH. These findings indicate that age-dependent effects on the expression of these genes were significantly influenced by postnatal overnutrition. No detectable interaction between postnatal overnutrition and age effects was observed on the expression of *Grin2a* (F_(1, 31)_ = 0.3186, *p* = 0.5765, Figure 4A), *Gabra1* (F_(1, 29)_ = 0.8504, *p* = 0.3640), *Slc12a2* (F_(1, 30)_ = 0.4786, *p* = 0.4944, Figure 4G), *Slc12a5* (F_(1, 29)_ = 0.02142, *p* = 0.8847, Figure 4H), *Abcc9* (F_(1, 29)_ = 1.429, *p* = 0.2415, Figure 4J) and *Kcnj8* (F_(1, 28)_ = 1.682, *p* = 0.2052, Figure 4K) in the ARH. Therefore, postnatal overnutrition induces long-term effects on the gene expression of N-methyl-D-aspartate receptor (*Gria-1*, *Grin2b* and *Grin*), GABA_B_ receptor (*Gabbr1* and *Gabbr2*) and K_ATP_ channels subunits (*Abcc8* and *Kcnj11*) in the ARH.

## 4. Discussion

The transition from prepubertal to pubertal stage is a critical stage of development. Our study revealed that this transition is characterized by a rise in excitatory and inhibitory transmission to ARH LepR-expressing neurons in female mice. The excitatory transmission was further increased by postnatal overnutrition, whereas the amplitude of inhibitory currents to ARH LepR-expressing cells was reduced in SL mice, suggesting that postnatal overweight exerts potent effects on ARH neurons activity. Postnatal overnutrition also contributes to the modulation of genes coding for some N-methyl-D-aspartate, GABA_B_ and K_ATP_ channel subunits in the ARH. Because the consequences of postnatal overnutrition were still observed in adult animals, our findings contribute to the literature by suggesting that postnatal overnutrition exerts long-term effects on ARH cellular excitability, as well changes in the transcriptional levels of selected genes.

Age-dependent synaptic plasticity has been described in ARH neurons, as well as in neurons of the paraventricular nucleus of the hypothalamus [16,17,21,34,35]. For example, ARH kisspeptin neurons exhibit a lower presynaptic inhibitory tone after puberty onset [35]. While, NPY or POMC-expressing neurons exhibit increased inhibitory synaptic transmission with age [16,17,21]. Our data are in accordance with the NPY and POMC findings because we observed that ARH LepR-expressing cells also exhibited increased spontaneous inhibitory transmission in the prepubertal to pubertal transition. Although we cannot assure that kisspeptin cells were not recorded among the LepR-expressing cells that were recorded, the co-localization between kisspeptin and LepR is observed only after sexual maturation development [36]. Therefore, the previously described changes in spontaneous activity plasticity of kisspeptin neurons [35] do not seem to contribute to our current findings. In the present study, age also contributed to increased excitatory tone to ARH LepR-expressing cells. Such effect has not been previously identified when kisspeptin or NPY-expressing neurons were recorded in the ARH [16,35]. In the present study most of recordings were performed in LepR-expressing cells located in the ventromedial part of the ARH, as mentioned. NPY/AgRP cells were probably recorded in abundance because this cell population is enriched in this region, whereas POMC neurons are concentrated more laterally in the ARH and most of kisspeptin neurons do not colocalize with LepR-expressing cells in adults [36]. Thus, although our recordings may have included different neuronal populations, the AgRP phenotype likely prevailed. Differences in the experimental approaches, including the sex of animals recorded, may explain the divergence between our data and the previous study [16].

In agreement with former studies that indicated an influence of body weight on synaptic plasticity [16,18,19,20,21,23], we observed that postnatal overnutrition further increases the presynaptic excitatory transmission to ARH LepR-expressing cells, beyond the rise already observed with puberty. Suggesting that early weight gain affect other brain nuclei which send projections to ARH LepR-expressing neurons. Local neural circuits in the ARH, including inhibitory projections mostly from non-AgRP GABAergic neurons to POMC cells, are part of the feeding circuitry [37]. Recent studies also disclosed that ARH neurons receive inputs from different hypothalamic neuronal populations. These circuits include excitatory projections from the paraventricular nucleus of the hypothalamus to AgRP neurons, and from neurons of the ventromedial nucleus of the hypothalamus to POMC cells [38,39]. Additionally, both AgRP and POMC neurons receive inhibitory inputs from GABAergic neurons located in the dorsomedial nucleus of the hypothalamus [40,41,42]. Therefore, different signaling pathways may have contributed to the present findings. The amplitude of postsynaptic inhibitory currents to ARH LepR-expressing cells was reduced in SL mice. Importantly, inhibitory currents recordings were not performed in the presence of glutamate ionotropic receptors antagonists or sodium channel blocker tetrodotoxin. Therefore, we cannot discard the fact that excitatory transmission may have contributed to the present findings. Additionally, we were unable to determine whether differences in sIPSC occur specifically at synapses. Nevertheless, the observed effects persisted in adult animals, even though body weight, fat mass and serum leptin levels become normal in adult mice raised in SL. Thus, increased serum leptin levels at the prepubertal stage may have contributed to the observed effects of SL on synaptic transmission. This idea is in accordance with the neurotrophic actions of leptin restricted to a specific neonatal window [6,7,14]. However, increased excitatory tone to NPY cells and increased inhibitory transmission to POMC neurons were also observed when cells were recorded from hypothalamic slices obtained from adult *ob/ob* mice in comparison to wild type animals [18], suggesting that the changes observed in our study could also be leptin-independent. It is out of the scope of the present investigation to determine whether there is another overweight- or age-dependent factor that may influence ARH plasticity.

Synaptic transmission via glutamate or GABA are critical for the hypothalamic circuitry regulating energy balance and the steroidal feedback control of GnRH secretion [20,43,44,45,46]. The effect of glutamate transmission in AgRP neurons seems to be required for the modulation of the feeding behavior [20,43]. The genetic ablation of *Grin* or *Grin2b* from AgRP cells, for example, but not from POMC cells, demonstrates that recruitment of these glutamatergic subunits in AgRP cells are required for body weight homeostasis [20,43]. However, the conditional deletion of *Grin2b* from AgRP neurons in *ob/ob* mice leads to a complete prevention of hyperglycemia-induced by the lack of leptin signaling, independently of changes in body weight and food intake [43]. Therefore, it will be of considerable interest to test in the future whether the observed effects of postnatal overnutrition on genes coding for glutamatergic receptors in the ARH favor the development of metabolic disorders in adult SL mice.

The NPY/AgRP and POMC neurons also link changes in energy balance to the regulation of the hypothalamic-pituitary-gonadal (HPG) axis [47,48]. However, how those ARH cells modulate the HPG axis activity is still a matter of investigation. The NPY/AgRP neurons exhibit direct inhibitory synaptic connections with kisspeptin neurons [48]. Therefore, we postulated that increased inhibitory transmission to ARH LepR-expressing cells contributes to the downregulation of inhibitory tone onto HPG components, which is an important event that contributes to puberty onset [16,35,45,49]. Furthermore, it is known that postsynaptically activation of GABA_B_ receptor may lead to the inhibition of GABA secretion [50]. Considering that postnatal overnutrition induces anticipation of sexual maturation, as displayed by individuals that exhibit higher serum leptin levels at prepubertal stage [25,51,52,53,54], GABA_B_ differential expression due to postnatal overnutrition may account for early sexual maturation. However, this hypothesis needs to be further investigated.

The differential expression of genes coding for K_ATP_ channel subunits has been previously postulated to act as a molecular gatekeeper for the maturation of feeding circuits [15,23]. While K_ATP_ channel activation can be induced in POMC cells from prepubertal mice, functional K_ATP_ channel is only found in mature NPY neurons in the ARH [15,55,56]. However, it has been demonstrated that the expression of genes coding for K_ATP_ channel subunits are very low in the ARH of prepubertal mice and increases with age [15,23]. Our data diverge from those previous investigations [15,23] because we observed that mRNA coding for most of K_ATP_ channel subunits were downregulated by age. A further study will be necessary to understand whether the chosen methodological approach to detect gene expression may explain the observed divergence between the studies. Nevertheless, while postnatal undernutrition suppresses the genes coding for the K_ATP_ channel subunits Sur2, Kir6.1 and Kir6.2 at postnatal day 30 [23], our data show that postnatal overnutrition, in turn, led to increased expression of genes coding for Sur1 (*Abcc8*) and Kir6.2 (*Kcnj11*) at prepubertal stage. Therefore, Kir6.2 seems to be the predominant subunit requested in the ARH due to metabolic demands as previously suggested [57]. Whether sex differences further contribute to the differential expression of genes coding for K_ATP_ channel subunits due to postnatal overweight gain needs to be further investigated. There are a variety of other receptor subunits that have not been investigated in the present study which can also contribute to the effects on ARH cell synaptic transmission. We believe that the differential gene expression due to postnatal overnutrition potentially contributes to the observed effects on synaptic transmission to ARH LepR-expressing cells. However, differential gene expression and changes in electrical activity are not necessarily correlated. It will be of interest to determine whether the observed mRNA expression variations due to postnatal overnutrition are accompanied by changes in protein levels either in whole cell or specifically at postsynaptic densities. Importantly, other central effects of an adipocyte-derived factor, such as leptin, are probably being affected by the observed alterations. Different from feeding behaviors which are not expected to be affected by leptin levels before the fourth week of age [5], modulation of genes coding for the N-methyl-D-aspartate, GABA_B_ or K_ATP_ channels subunits in the ARH due to postnatal overnutrition may, for example, contribute to early puberty onset or predispose individuals for other metabolic diseases. This hypothesis requires further investigation.

## 5. Conclusions

We contribute to the field demonstrating that the transition from prepubertal to pubertal stage is characterized by a rise in both excitatory and inhibitory transmission to ARH LepR-expressing neurons. Age-dependent effects on the expression of genes coding for ionotropic glutamate, GABAergic, Na^+^-K^+^-Cl^−^ cotransporter and K_ATP_ channel subunits were mostly downregulated in adult mice. A further study will be required to determine the exact age in which the increase of excitatory and inhibitory transmission to ARH LepR-expressing cells occurs and whether sex differences can contribute to differences in the development of the ARH plasticity. Importantly, we further demonstrated that increased adiposity during the postnatal period induces permanent effects on neural activity. In addition to the knowledge that K_ATP_ channel subunits contribute to the formation of feeding circuitry [23], we demonstrated that a broad range of genes are affected by postnatal overnutrition. Whether postnatal overnutrition is able to affect synaptic transmission into other brain areas or predispose individuals to neurodegenerative disorders deserves further investigation.

## Figures and Tables

**Table 1:** Primer sequences.

Gene	Forward Primer (5′–3′)	Reverse Primer (5′–3′)
*Abcc8*	5′-gtcctgaggcaatacctggg	gtcttctggaacagcagcct
*Gabbr1*	atactgcacgccgttctgag	tcccggagcatctgtagtca
*Gabbr2*	gttgtactcgccgaccttca	agtcacgggtcaagttgtgtt
*Gabra1*	gtccagcagtcggtccaaaa	agcacactgtcgggaagaag
*Gapdh*	gggtcccagcttaggttcat	tacggccaaatccgttcaca
*Gria-1*	ccaatcccagccctccaatc	cggaagtaaggacaagaccagt
*Grin1*	cccggtgctcgtgtcttt	cgtgaacgtgtggaggaaga
*Grin2a*	ccatctcaccgtcaccaacaa	aattgctctgcagaagggctc
*Grin2b*	accaaatcgctttgccgatg	Ttacaaccggtgcctagctg
*Kcnj8*	cgcagacgtgaatgacctga	catggagaagagtggcctgg
*Kcnj11*	cctcctctctcgagtacggt	gctgtcccgaaagggcatta
*Slc12a2*	cagaaagatgaggaagaggatggc	ggcacaatagggcctttggat
*Slcl2a5*	gaataaaggccccagtcccg	aagttttcccactccggctt

**Table 2:** Postnatal overnutrition model validation.

Body Weight (g)	Prepubertal	Pubertal	Adult	*p*-Value
Control	5.0 ± 0.2 (*n* = 15)	14.7 ± 0.4 (*n* = 12)	18.6 ± 0.3 (*n* = 14)	age: F_(2, 59)_ = 790.0, *p* < 0.0001
SL	8.2 ± 0.3 (*n* = 8) ****	17.3 ± 0.3 (*n* = 8) ****	19.3 ± 0.3 (*n* = 8)	litter size: F_(1, 59)_ = 62.5, *p* < 0.0001
				Interaction: F_(2, 59)_ = 7.7, *p* = 0.001
**Fat Pad (g)**				
Control	0.02 ± 0.002 (*n* = 8)	0.05 ± 0.003 (*n* = 7)	0.06 ± 0.007 (*n* = 4)	age: F_(2, 37)_ = 34.9, *p* < 0.0001
SL	0.05 ± 0.002 (*n* = 8) ****	0.06 ± 0.004 (*n* = 7)	0.06 ± 0.002 (*n* = 9)	litter size: F_(1, 37)_ = 31.1, *p* < 0.0001
				Interaction: F_(2, 37)_ = 8.9, *p* = 0.007
**Serum Leptin (pg/mL)**				
Control	1.3 ± 0.2 (*n* = 8)	0.4 ± 0.04 (*n* = 8)	0.2 ± 0.02 (*n* = 8)	age: F_(2, 42)_ = 54.7, *p* < 0.0001
SL	5.0 ± 0.7 (*n* = 8) ****	0.3 ± 0.04 (*n* = 8)	0.3 ± 0.02 (*n* = 8)	litter size: F_(1, 42)_ = 21.8, *p* < 0.0001
				Interaction: F_(2, 42)_ = 22.3, *p* < 0.001
**Sexual Maturation**	**Vaginal Opening (days)**	**First Estrus (days)**		
Control	33.7 ± 1.3 (*n* = 14)	43.4 ± 1.6 (*n* = 11)		
SL	29.8 ± 0.5 (*n* = 18) **	37.3 ± 1.0 (*n* = 12) **		

Mean ± SEM. Body weight, subcutaneous fat pad and serum leptin levels were evaluated by unpaired two-way ANOVA, followed by the Bonferroni post hoc test. Sexual maturation was evaluated by the Mann-Whitney test. ** *p* < 0.005, **** *p*< 0.0001 versus control group.
